# Hydronephrosis in Children Caused by Lower Pole Crossing Vessels—How to Choose the Proper Method of Treatment?

**DOI:** 10.3389/fped.2019.00083

**Published:** 2019-03-19

**Authors:** Marcin Polok, Krystian Toczewski, Dominika Borselle, Wojciech Apoznański, Diana Jędrzejuk, Dariusz Patkowski

**Affiliations:** ^1^Department of Pediatric Surgery and Urology, Wroclaw Medical University, Wroclaw, Poland; ^2^Department of Endocrinology, Diabetology and Isotope Therapy, Wroclaw Medical University, Wroclaw, Poland

**Keywords:** crossing vessel, vascular hitch, pyeloplasty, ureteropelvic junction obstruction, hydronephrosis, children, pediatric

## Abstract

**Objectives:** Assessment of the efficacy of intraoperative diagnosis between extrinsic and intrinsic UPJO in children. Assessment of the efficacy of laparoscopic vascular-hitch procedure in UPJO caused by lower pole crossing vessels (CV).

**Materials and Methods:** Between 2008 and 2017, 47 laparoscopic procedures were performed with the CV discovered intraoperatively. CV were translocated cephalad, and the UPJ was carefully inspected. The Chapman's vascular hitch procedure was accomplished in the case of decreasing sizes of the pelvis and clear, visible peristalsis of the UPJ (31 patients). In the other cases, Anderson–Hynes (A-H) pyeloplasty with posterior translocation of the CV was performed (16 patients).

**Results:** The median age at operation was 6 years (range 1–16) in VH and 6 years (range 2–17) in A-H (*p* = 0.4635). Prenatal dilatation of kidney was diagnosed in 18.7% of VH and 10% of A-H cases (*p* = 0.5474). Success was achieved in 16 (100%) patients in the A-H and in 29 (93.54%) in the VH groups. Two patients (6.5%) in VH required repeated surgery because of a misdiagnosed intrinsic obstruction. Median operation time in VH was 80 min (range 40–105) and was 105 (range 70–225) in A-H (*p* < 0.05).

**Conclusions:** The intraoperative selection based on intraoperative pelvis and UPJ appearance after vessel transposition is sufficient in majority of cases. Laparoscopic vascular hitch seems to be effective and safe procedure, but can only be performed on carefully selected patients. In case of misdiagnosis, reoperation is possible with the same laparoscopic access.

## Introduction

The classical operative procedure for ureteropelvic junction obstruction (UPJO) in children is dismembered pyeloplasty, which was described by Anderson and Hynes in 1949 (1, 2). In the same year Hellstroem presented a technique, applied in the case of crossing vessels that causes mechanical obstruction of the UPJ (3). The “vascular hitch” procedure means cephalad translocation of the CV and fixing it to the tissues around the kidney. In the next years, many different modifications of this technique appeared, i.e., the most popular one was introduced by Chapman (4). The author recommended fixing the CV in a tunnel made of the pelvis, away from the UPJ. This technique enabled the surgeon to save the CV covered by pelvic tissues from being punctured with needle and any complications associated with vascular damage. Vascular hitch procedures in comparison to dismembered pyeloplasties are less technically demanding and shorten the operation time (5). The proper choice of operative treatment for UPJO with CV is very important, as simultaneous intrinsic obstruction of the UPJ could be overlooked. In this article, we present our experience in children with transperitoneal laparoscopic approach for the treatment of hydronephrosis caused by CV.

## Aim

Assessment of the efficacy of intraoperative diagnosis between extrinsic and intrinsic UPJO with CV in children. Assessment of the efficacy of the laparoscopic vascular-hitch procedure in UPJO caused by lower pole crossing vessels.

## Materials and Methods

Between 2008 and 2017 in the Department of Pediatric Surgery and Urology in Wroclaw, 47 laparoscopic hydronephrosis operations with intraoperatively diagnosed lower pole crossing vessels at the level of UPJ were performed. Retrospective analysis of the medical history was carried out. As a qualification for surgical treatment in all patients, ultrasound of the kidneys and diuretic renography were scheduled. In questionable cases, CT scans were done. The lower pole CV before surgery were suspected in 18 (38.3%) of the patients on the ultrasound examination and in 30 (63.8%) in computed uro-tomography. In all cases, an enema of the large intestine was performed 24 h before surgery. Laparoscopic, transperitoneal technique with three 5 mm or two 3 mm and one 5 mm ports was used. Access to the left kidney was achieved through a window in the colonic mesentery. On the right side, the colon was mobilized. The UPJ was released by blunt dissection and an electrocautery hook. Detected crossing vessels were translocated cephalad, and the UPJ was carefully inspected. In case of a decreasing pelvis and a clear, visible peristalsis of the UPJ, the Vascular Hitch with Chapman modification was performed. The CV was fixed with 2–3 sutures in a tunnel made from the pelvis, far away from UPJ. This procedure was done in 31 children (VH group). In situations when the pelvis did not decrease or/and there were visible stenosis or no clear peristalsis of the UPJ after the release and translocation of the CV the Anderson–Hynes dismembered pyeloplasty with posterior translocation of the CV was performed. This procedure was used in 16 patients (A-H group). Three patients were operated upon before by using open surgery: one of them twice in another center, and two patients in our department. In these cases, CV was not discovered during the primary surgery. The median follow-up was 4 years (range 0.5–6) in the VH group and 3 years (range 0.5–6) in the A-H group.

To compare numerical samples, both the Student's test and the non-parametric unpaired Wilcoxon test (known also as the Mann-Whitney test) were utilized. The choice of the most suitable test was made based on responses of the Ljung-Box test (independence within each sample), the Shapiro-Wilk test (normality of distributions in each sample), and the F test (equality of variances in two samples). To compare categorical (dichotomic) samples, the test for equal proportions was used. The statistical analysis was performed in R, the language and environment for statistical computing.

## Results

Demographic data comparing two groups of patients are presented in Table 1. There was no statistically significant difference in any parameter except the side of the kidney. Success was achieved in 16 (100%) patients in the A-H group and 29 (93.54%) in the VH group. Two patients (6.46%) in the VH group required reoperation because of undiagnosed intrinsic obstruction. The retrospective analysis of videos of surgeries in these two patients showed incorrect assessment of renal pelvis emptying after relocation of CV. Both were reoperated using the same transperitoneal laparoscopic access without any technical difficulties. Secondary JJ stent insertion was needed in one patient (3.2%), but after removal of the stent, there were no symptoms of hydronephrosis. There was no need for conversion in any case. The median operative time in the VH group was 80 min (range 40–105 min) and 105 min (70–225 min) in the A-H group (*p* < 0.05). Outcomes in both groups of patients (VH and A-H) are given in Table 2. The limitation of the study is that it was performed retrospectively.

**Table 1:** Demographic data comparing two groups of patients.

	**Method of treatment**		***p*-value**
	**VH (Vascular-Hitch)**	**A-H (Anderson-Hynes)**	
Number of pts	31	16	
Age in years—median (range)	6 (1–16)	6 (2–17)	0.4635
Gender (M—male, F—female)	M−18, F−13	M−11, F−5	0.4751
Side (R—right, L—left)	R−7, L−24	R−7, L−9	0.0264
Prenatal diagnosis [yes/all (%)]	3/16 (18.7%)	1/10 (10%)	0.5474
Symptoms yes—pain [yes/all (%)]	16/20 (80%)	7/11 (63.63%)	0.3191
Ultrasound-dilatation of kidney pelvis in AP (cm) before surgery – median (range)	4,5 (2.5–7)	3 (1.3–6)	0.1944
Diureric renography before surgery-median (range)	41% (23–53)	42% (14–57)	0.9548
Follow up in years—median (range)	4 (0.5–6)	3 (0.5–9)	0.9730

**Table 2:** Outcomes of vascular-hitch-group patients and anderson-hynes-group patients.

	**VH (Vascular-Hitch)**	**A-H (Anderson-Hynes)**
Number of pts (%)	31 (100%)	16
Redo surgery	2 (6.5%)	0
Redo JJ	1 (3.2%)	0
Symptoms resolution	29 (93.54%)	8 (100%)
Ultrasound—improvement	29 (93.54%)	16 (100%)
DR—improvement or stable function	11 of 11 (100%)	11 of 11 (100%)
Success rate	29 (93.54%)	16 (100%)

## Discussion

A very important role in diagnosis of patients with UPJO is played by a carefully gathered clinical history. In most children with CV, there is no history of hydronephrosis in the neonatal period, with a frequency of 75–100% (5–8). In our material dilatation of the kidneys during pregnancy and in neonatal period had 18.7% of children in the VH group and 10% in the A-H group (0.5474). Furthermore, the typical clinical picture for these patients is presenting with colicky flank pain, which is sometimes associated with vomiting. In the literature, the incidence of colic pain in pure extrinsic UPJO is given as 71.8–100% (5, 8, 9). In our patients, in the VH group it happened in 80% but was only in 63.6% of children in the A-H group. The incidence of CV causing obstruction of the UPJ in children increases with age. CVs are very rarely noticed in newborns and infants. According to the literature, the average age of patients with a CV is between 7 and 11 years and is statistically higher than in patients with pure intrinsic obstruction (8–12). In our material, the median age at surgery was 6 years in both groups.

The key to success is adequate patient's selection, but this faces some challenges. It can be difficult task to whether the CV are an incidental finding or play a significant role in the obstruction (13). In our material the decision whether to use the vascular hitch procedure or dismembered pyeloplasty was taken intraoperatively. The UPJ was carefully inspected after the cephalad translocation of crossing vessel. In the case of decreasing or emptying renal pelvis and a clear, visible peristalsis of the UPJ, the Chapman procedure was performed. Otherwise or in case of apparent UPJ stenosis Anderson-Hynes pyeloplasty with posterior translocation of the crossing vessel was done. Our intraoperative technique to select patients seems to be very successful. Based on it, a 95.74% overall success rate was observed (100% in A-H and a 93.54% in VH). Only in two patients after VH there was a need for reoperation. The retrospective analysis of recorded videos revealed incorrect assessment of renal pelvis emptying after relocation of CV. This could be the reason for failure. In both cases there was no prenatal history of hydronephrosis. There were no other complications. In other studies surgeons using the similar technique of laparoscopic intraoperative selection between intrinsic and extrinsic obstruction reported success rate between 96 and 100% (14–18). However, they had much lower number of cases ranged between 8 and 19. Villemagne et al. on the cohort of 70 children using laparoscopy in 42 cases and robotic-assisted surgery in 28 cases reached a success in 67 (96%) (13). They concluded, that purely extrinsic obstruction can be deduced from a dramatic decrease in the renal pelvis size between the beginning and the end of the PUJ mobilization, and by observing pelvic emptying. With these findings at surgery, in the context of a child with the typical clinical picture of intermittent hydronephrosis, no prenatal history and preserved renal function, the possibility of associated intrinsic stenosis is low.

The advantage of the laparoscopic vascular hitch procedure over dismembered pyeloplasty is the absence of the need to open the collecting system, leaving the PUJ intact, avoiding the technical challenge of pelviureteric anastomosis. It is a quite simple technique compared to pyeloplasty (5). It can also be performed by less experienced surgeons in minimally invasive surgery. The time of the surgery is shorter than in laparoscopic dismembered pyeloplasty. In our material, in the VH group, the mean operative time was 80 min (range 40–105 min) and 105 min (70–225 min) in the A-H group with statistical difference (*p* < 0.05) however the operating surgeon had a great experience in suturing.

Because of the risk of overlooking a simultaneous intrinsic obstruction some authors recommend additional, intraoperative diuretic-test. In a multicentre study, Esposito et al. used this technique in 51 patients with CV (5). In the laparoscopy before the vessels mobilization, they administered a bolus of normal saline, followed by furosemide. In all patients, they observed symptom resolution and no need for repeat surgery. Miranda et al. performed laparoscopic vascular hitch with modified Whitaker test in 4 of 11 children. A fine needle was inserted percutaneously into the renal pelvis and the ureteral opening pressure was evaluated three times using a water column device. They assumed that if the opening pressure was lower than 14 cm of water, then the junction was considered to be unobstructed. None of their four patients required reoperation (19). Because of the small number of patients, this study is less valuable. Parente et al. calibrated UPJ using a high-pressure balloon inserted by cystoscopy and inflated at the UPJ level in 10 patients with suspected CV (20). They considered intrinsic obstruction to be present where a “waist” was observed at the UPJ on inflation of the balloon and a laparoscopic dismembered pyeloplasty was performed. When no “waist” was observed, laparoscopic vascular hitch was performed. They found no intraoperative and postoperative complications using this technique. In our opinion, there is no need for additional, invasive procedures like the Whitaker test. The puncture of the pelvis causes the risk of complications like urine leakage and urinoma formation after surgery. The classical intraoperative, diuretic test with furosemide could be taken into consideration. It is simple to perform, does not extend the surgical time and maybe could reduce the risk of misdiagnosis.

The intraoperative selection based on intraoperative pelvis and UPJ assessment after vessel transposition is sufficient in majority of cases. Laparoscopic vascular hitch seems to be effective and safe procedure but can only be performed on carefully selected patients. In case of misdiagnosis, reoperation is possible with the same laparoscopic access.

## Data Availability

All datasets generated for this study are included in the manuscript and/or the supplementary files.

## Ethics Statement

The consent was obtained from all participants both informed and written. Ethics board approval number is KB-341/2018 (Ethics Committee by Medical University, University of Wrocław).

## Author Contributions

MP and DP contributed conception and design of the study. DB, KT, MP, and DJ organized the database. MP performed the statistical analysis. MP wrote the first draft of the manuscript. WA and DP wrote sections of the manuscript. All authors contributed to manuscript revision, read, and approved the submitted version.

### Conflict of Interest Statement

The authors declare that the research was conducted in the absence of any commercial or financial relationships that could be construed as a potential conflict of interest.
